# Network localization of functional and structural correlates of apathy in Parkinson’s disease

**DOI:** 10.3389/fnsys.2026.1724421

**Published:** 2026-03-03

**Authors:** Hu-Cheng Yang, Si-Yu Gu, Hai-Hua Sun, Yuan-Ying Song, Feng-Mei Zhang, Zhen-Yu Dai, Ping-Lei Pan

**Affiliations:** 1 Department of Radiology, Affiliated Hospital 6 of Nantong University, Yancheng Third People’s Hospital, Yancheng, China; 2 Department of Radiology, Binhai Maternal and Child Health Hospital, Yancheng, China; 3 Department of Neurology, Affiliated Hospital 6 of Nantong University, Yancheng Third People’s Hospital, Yancheng, China

**Keywords:** apathy, frontoparietal network, functional connectivity network mapping, network localization, Parkinson’s disease, subcortical network, ventral attention network

## Abstract

**Background:**

Apathy is a prevalent and debilitating neuropsychiatric syndrome in Parkinson’s disease (PD). While numerous functional and structural brain studies have investigated the neural correlates of PD with apathy (PD-A), their findings have often been inconsistent. Network neuroscience suggests that such a syndrome may be best understood as disruptions of distributed brain networks.

**Methods:**

We conducted a systematic review to identify whole-brain studies reporting functional or structural alterations in patients with PD-A compared to those without apathy (PD-NA), or studies correlating apathy severity. Significant peak coordinates (195 foci from 24 studies) were integrated using functional connectivity network mapping (FCNM), leveraging resting-state functional magnetic resonance imaging from 1,093 healthy Human Connectome Project (HCP) participants. We quantified spatial overlap between the PD-A-associated network and canonical brain networks.

**Results:**

The FCNM analysis revealed that the spatially diverse brain regions previously reported in the PD-A literature converged onto a common functional connectivity network. This network predominantly involved the bilateral inferior frontal gyrus, bilateral anterior insula, bilateral dorsolateral prefrontal cortex, bilateral caudate nucleus, and bilateral thalamus. The PD-A associated network showed the highest spatial overlap with the ventral attention network (VAN; 34.05%), subcortical network (28.47%), and frontoparietal network (FPN; 24.89%). Robustness analyses confirmed these findings.

**Conclusion:**

Brain functional and structural correlates of apathy in PD converge on distributed networks involving the VAN, FPN, and subcortical circuits. Our network localization approach offers a unifying neurobiological framework for apathy in PD, potentially reconciling previous inconsistencies and informing the development of network-targeted interventions.

## Introduction

Apathy, characterized by diminished motivation, goal-directed behavior, and emotional responsivity, is a common neuropsychiatric syndrome in Parkinson’s disease (PD) (den Brok et al., 2015; Béreau et al., 2023), with reported prevalence rates varying widely from 12 to 70% across different studies (den Brok et al., 2015; Mele et al., 2019). This syndrome profoundly impairs daily functioning (Leroi et al., 2012; Liu et al., 2024) and overall quality of life (Plant et al., 2024; Benito-León et al., 2012), and also serve as a predictor of dementia (Dujardin et al., 2009). Furthermore, it poses a significant burden on caregivers, who often perceive it as one of the most distressing symptoms of the disease (Leroi et al., 2012; Leiknes et al., 2010). While traditionally linked to dysfunction within frontostriatal circuits (Hamada et al., 2021; Morris et al., 2023), particularly the mesocorticolimbic dopaminergic pathways (Prange et al., 2022), the precise pathophysiology of PD with apathy (PD-A) remains incompletely understood.

Neuroimaging studies have sought to identify structural or functional abnormalities in isolated brain regions associated with apathy, including the prefrontal cortex (Maggi et al., 2024), anterior cingulate cortex (Prange et al., 2022), basal ganglia (Wen et al., 2022), and limbic system (Luo et al., 2022). However, these findings have been markedly inconsistent, with considerable heterogeneity in both the neuroanatomical loci and the extent of brain changes. This variability is widely attributed to a combination of factors, such as methodological differences among studies, small sample sizes, and the multifaceted nature of apathy itself (Luo et al., 2022; Levy and Dubois, 2006). Consequently, this heterogeneity has limited the elucidation of the precise brain-behavior relationships underlying apathy in PD, thereby hindering the development of reliable biomarkers and targeted interventions.

Recent advances in network neuroscience have prompted a paradigm shift from localizing symptoms to single regions towards a framework that conceptualizes them as disorders of large-scale, distributed brain networks (Peng et al., 2024; Darby et al., 2019). Mounting evidence supports this view, suggesting that neuropsychiatric manifestations, such as apathy, arise not solely from damage to a single brain area, but rather reflect dysfunction within interconnected brain networks (Tian et al., 2025; Alfano et al., 2021). Network localization techniques, such as lesion network mapping (Ding et al., 2023), activation network mapping (Peng et al., 2022), and functional connectivity network mapping (FCNM) (Mo et al., 2024), integrate focal neuroimaging findings (e.g., sites of atrophy, hypometabolism, or dysfunction) with large normative connectomes to identify common brain networks underlying diverse clinical symptoms (Fox, 2018). In contrast to conventional coordinate-based meta-analytic methods (e.g., activation likelihood estimation, ALE) that primarily identify spatial convergence, FCNM aims to test whether spatially heterogeneous abnormalities can be localized to a shared distributed network (Mo et al., 2024). While such network-based approaches have successfully mapped the neural circuits of complex behaviors and neuropsychiatric phenomena across a range of neurological and psychiatric conditions (Fox, 2018; Xu et al., 2024; Schaper et al., 2023; Zhang et al., 2024; Tetreault et al., 2020; Boes et al., 2015; Yang et al., 2025; Song et al., 2025), including recent applications to the general pathophysiology of PD (Song et al., 2025) and specific non-motor symptoms like impulse control disorders (Yang et al., 2025), it remains largely unknown whether the neuroanatomical substrates of PD-A can be similarly elucidated from a network perspective.

Therefore, the primary objective of this study was to map the brain network associated with PD-A by integrating previous neuroimaging markers of brain abnormality with a large-scale normative functional connectome. Specifically, we hypothesized that diverse and spatially heterogeneous brain regions previously implicated in PD-A would converge onto a common network. By employing network localization techniques, we sought to test this hypothesis and determine whether a unified network can account for these seemingly disparate anatomical findings. Identifying such a network could advance the neurobiological model of PD-A and inform the development of targeted, network-based interventions.

## Methods

### Literature search and study selection

Following the PRISMA guidelines (Page et al., 2021), we systematically searched PubMed, Embase, and Web of Science for studies reporting functional or structural alterations related to PD-A, up to April 10, 2025. (A detailed list of the search terms is provided in Supplementary material). We further identified relevant studies by manually screening the references of review articles and meta-analyses. The study selection process is detailed in Supplementary Figure S1.

Studies were included if they met the following criteria: (a) published in a peer-reviewed English-language journal as an original article; (b) involved whole-brain comparison [gray matter volume (GMV), task-related activation, and resting-state activity] between PD-A patients and PD-NA patients or analyzed the correlation between apathy severity and neuroimaging metrics; (c) reported results as coordinates in Talairach or Montreal Neurological Institute (MNI) space. For longitudinal studies, only baseline data were included.

Exclusion criteria were as follows: (a) non-original studies (e.g., review, meta-analysis, meeting abstract); (b) no coordinate system reported; (c) studies employing region-of-interest analyses; (d) studies involving animal experiments. To avoid data duplication from overlapping patient samples across different publications, only the study reporting the largest sample size and most comprehensive information was retained for analysis. The corresponding author of each included study was contacted via email when additional information was required. Two investigators (H. C. Y. and S. Y. G.) independently performed literature search and selection and data extraction. Any discrepancies were discussed with another investigator (P. L. P.) until they were resolved.

### Normative resting-state functional magnetic resonance imaging data acquisition and preprocessing

We used a normative functional connectome derived from 1,093 healthy adults [594 female; mean (SD) age 28.78 (3.69) years, range 22–37] from the HCP 1200 Subjects Data Release (HCP-S1200) (Van Essen et al., 2012) following standard quality control. Individuals in the cohort were between the ages of 22 and 37. Participants in the HCP were free of MRI contraindications, current psychiatric or neurological disorders, recent use of psychiatric medication, pregnancy, and a history of head trauma. The specific demographics of the analyzed sample can be found in Supplementary Table S1. All coordinates from prior studies were converted to MNI space using established tools^1^ if originally reported in Talairach space.

In this study, we utilized resting-state fMRI data from the HCP (Van Essen et al., 2012), obtained using a 3 T Siemens Skyra system. This dataset was considered appropriate for comprehensive analysis due to its high quality and large sample size. The specific acquisition parameters can be found in Supplementary Table S2. Individuals were excluded from the study if their imaging data did not pass quality assessments, such as the detection of artifacts or incomplete brain coverage.

The resting-state fMRI data underwent preprocessing with SPM12 software^2^ and the Data Processing and Analysis for Brain Imaging (DPABI) toolbox.^3^ The initial 10 volumes of each participant’s scan were discarded to facilitate signal equilibration and adaptation to scanner noise. Slice acquisition timing disparities in the remaining volumes were corrected, and subsequently, motion correction was performed. Head motion parameters for each volume were determined by calculating translational displacements in each direction and angular rotations around each axis. All participant data met the specified motion thresholds, with maximal translational or rotational motion parameters below 2 mm or 2°. Furthermore, framewise displacement was computed as a measure of head motion between volumes. Regression analysis was conducted to account for nuisance effects, incorporating covariates such as linear drift, estimated Friston-24 motion parameters, timepoints with excessive motion (framewise displacement > 0.5 mm), and signals from global mean, white matter, and cerebrospinal fluid. In line with previous network mapping studies that seek to improve system-specific correlations, global signal regression was incorporated into the preprocessing pipeline. The datasets were bandpass filtered to preserve frequencies ranging from 0.01 to 0.1 Hz. Initially, spatial normalization began with co-registering each participant’s T1-weighted structural image with their average functional image. These structural images were then segmented and normalized to MNI space. Each filtered functional volume was then normalized to MNI space. Subsequently, all functional data were spatially smoothed using a Gaussian kernel (FWHM = 6 × 6 × 6 mm^3^).

### FCNM analysis

We utilized the FCNM approach to determine if the reported brain regional alterations in PD-A map onto a common brain network. For each identified contrast in the literature search, spheres with a 4-mm radius were centered at the reported coordinates of significant difference. These spheres were then merged to form a contrast-specific seed mask, hereafter termed the “contrast seed.” Subsequently, functional connectivity (FC) maps were generated for each participant by deriving the FC from each contrast seed to the entire brain based on preprocessed resting-sate fMRI data from 1,093 healthy HCP participants. The procedure comprised calculating Pearson’s r between the average time course of the seed for the contrast and the time course of all remaining brain voxels, subsequently applying a Fisher Z-transform to normalize the resultant correlation coefficients. Third, the participant-level FC maps (*N* = 1,093) were subjected to a voxel-wise one-sample t-test to identify brain regions consistently exhibiting positive FC with each contrast seed across the normative samples. Consistent with previous FCNM studies, we focused solely on positive FC, as the biological interpretation of negative FC remains under debate (Murphy et al., 2009; Murphy and Fox, 2017). After thresholding at *p* < 0.05, the group-level t-maps were corrected for multiple comparisons using a voxel-level false discovery rate (FDR) method and subsequently binarized. Finally, the binarized connectivity maps corresponding to all included contrasts were overlaid to form a network probability map. This map was then thresholded at 60% overlap (i.e., regions connected to at least 60% of the contrast seeds), a threshold validated in previous FCNM studies (Peng et al., 2022; Mo et al., 2024), to delineate the PD-A associated brain networks.

### Association with canonical brain networks

To enhance interpretability, we analyzed the spatial overlap between the identified PD-A-associated network and eight established canonical brain networks. The visual network, somatomotor network (SMN), dorsal attention network (DAN), ventral attention network (VAN), limbic network, frontoparietal network (FPN), and default mode network (DMN) are the seven cortical networks defined by Yeo et al. The Human Brainnetome Atlas was utilized to delineate the subcortical network. The subcortical network mainly includes the amygdala, hippocampus, basal ganglia (including the caudate nucleus, putamen, globus pallidus, nucleus accumbens), and thalamus. We quantified these spatial relationships by calculating the proportion of overlapping voxels between each PD-A-associated network and each canonical network, relative to the total number of voxels within the respective canonical network.

## Results

### Included studies and sample characteristics

Following the systematic literature search and screening process detailed in Supplementary Figure S1, a total of 24 studies (providing 195 foci) were included, comprising 1,206 patients with PD, of whom 606 were diagnosed with apathy (PD-A) and 600 were without apathy (PD-NA) (Remy et al., 2005; Reijnders et al., 2010; Alzahrani et al., 2016; Le Jeune et al., 2009; Thobois et al., 2010; Terada et al., 2018; Prange et al., 2019; Robert et al., 2012; Huang et al., 2013; Skidmore et al., 2013; Robert et al., 2014; Robert et al., 2014). Regarding the analytical approaches, 10 studies performed group comparisons, 10 studies conducted correlation analyses between apathy scores and neuroimaging metrics, and 4 studies utilized both methodologies. Imaging modalities included PET (*n* = 9) and MRI (*n* = 15), with 16 studies utilizing the MNI template and 8 studies utilizing the Talairach template for spatial normalization. Among the studies, 7 utilized structural MRI, focusing on GMV or GM density alterations; 8 employed resting-state fMRI to investigate changes in spontaneous brain activity using measures such as amplitude of low-frequency fluctuations, regional homogeneity, voxel-mirrored homotopic connectivity, and FC; and one study implemented task-based fMRI. Detailed sample and imaging characteristics of the included studies are summarized in Table 1.

**Table 1:** Sample and imaging characteristics of the studies included.

Study	Sample size (PD-A/-NA)	Mean age (PD-A)	Apathy assessment	Analysis	Comorbidity control	Medication/DBS status	Threshold	Scanner	Template	Measure	Foci
Remy et al. (2005)	20/0	59.8 (7.2)	AES	Correlation	Excluded for dementia; Apathy/anxiety not controlled for	Medication-OFF; No DBS	*P* < 0.05, uncorrected	PET	Talairach	Binding Potential	2
Reijnders et al. (2010)	55/0	62.0 (10.1)	AES/LARS/NPI-A	Correlation	Excluded for major depression/dementia	Medication status not specified; No DBS	*P* < 0.05, FDR corrected	3 T MRI	MNI	GMD	28
Alzahrani et al. (2016)	25/40	68.7 (8.4)	NPI-A	Comparison (PD-A-PD-NA)	Excluded for dementia and other neuropsychiatric comorbidities	Medication status not specified; No DBS	*p* < 0.001, corrected	1.5 T MRI	Talairach	GMV	9
Le Jeune et al. (2009)	12/0	57.4 (8.00)	AES	Correlation	Excluded for dementia and major psychiatric disorders	Medication-ON; Post-DBS	*p* < 0.005, corrected	PET	Talairach	Glucose metabolism	8
Thobois et al. (2010)	12/13	55.8 (4.9)	SAS	Comparison (PD-A-PD-NA)	Excluded for baseline apathy and mod-severe depression	Post-DBS; Drug withdrawal	*P* ≤ 0.001, uncorrected	PET	MNI	D2/D3 receptor availability	11
Terada et al. (2018)	40/0	64.7 (8.0)	FrSBE	Correlation	Excluded for dementia & depression	Medication-OFF; No DBS	*P* < 0.05, FWE corrected	1.5 T MRI	MNI	GMV	1
Prange et al. (2019)	14/13	62.5 (10.2)	LARS/SAS	Comparison	Excluded for dementia; depression as covariate	Drug-naïve	*P* < 0.05, FWE corrected	1.5 T MRI	MNI	GMV	3
Robert et al. (2012)	45/0	60.5 (7.8)	AES	Correlation	Excluded for depression and dementia	Medication-ON; No DBS	*P* < 0.005, uncorrected	PET	MNI	Glucose metabolism	6
Huang et al. (2013)	26/0	66.5 (1.4)	AES	Correlation	Excluded for dementia; depression or anxiety not excluded	Medication-OFF; No DBS	*P* < 0.001, uncorrected	PET	MNI	Glucose metabolism	6
Skidmore et al. (2013)	15/0	63 (9)	LARS	Correlation	Excluded for dementia; depression as covariates	Medication-OFF; No DBS	*P* < 0.005, uncorrected	3 T MRI	MNI	ALFF	8
Robert et al. (2014)	36/0	58.6 (7.3)	AES	Correlation	Excluded for dementia & major depression; subclinical depression controlled	Medication-ON; No DBS	*P* < 0.005, uncorrected	PET	Talairach	Glucose metabolism	8
Maggi et al. (2024)	48/287	60.98 (9.81)	UPDRS-I	Comparison (PD-A-PD-NA)	Not controlled for (depression and anxiety were measured as outcome variables)	Drug- naïve at baseline	*P* < 0.05, FWE corrected	3 T MRI	MNI	GMD	8
Robert et al. (2014)	44/0	56.3 (7.5)	AES	Correlation	Excluded for depression and dementia	Medication-ON; Pre-DBS	*P* < 0.005, uncorrected	PET	Talairach	Glucose metabolism	1
Auffret et al. (2017)	12/0	65.9 (7.2)	LARS	Correlation	Excluded for dementia	Medication-ON; No DBS	*P* < 0.001, uncorrected	PET	Talairach	Glucose metabolism	18
Shin et al. (2017)	10/12	73.8 (4.3)	AS	Comparison (PD-A-PD-NA) correlation	Excluded for dementia & depression	Medication-OFF; No DBS	*P* < 0.05, corrected	3 T MRI PET	MNI	Glucose metabolism GMV	21
Shen et al. (2018)	20/22	63.35 (8.52)	AS	Comparison (PD-A-PD-NA) correlation	Excluded for dementia; depression, and anxiety as covariates	Medication-OFF; No DBS	*p* < 0.01, corrected	3 T MRI	MNI	ALFF	5
Sun et al. (2020)	20/26	59.85 (8.92)	AS	Comparison (PD-A-PD-NA)	Excluded for dementia, depression, and anxiety	Drug- naïve	*P* < 0.05, corrected	3 T MRI	MNI	ALFF	4
Sun et al. (2020)	20/26	59.85 (8.92)	AS	Comparison (PD-A-D-NA) correlation	Excluded for dementia, depression and anxiety	Drug- naïve	*P* < 0.05, corrected	3 T MRI	MNI	ReHo	2
Xu et al. (2022)	28/19	65.1 (6.2)	AS	Comparison (PD-A-D-NA)	Excluded for dementia; Not controlled for depression/anxiety	Medication-OFF; No DBS	Voxel *P* < 0.01, cluster *P* < 0.05, corrected	3 T MRI	MNI	ALFF	1
Zhang et al. (2022)	26/27	57.92 (8.65)	AS	Comparison (PD-A-PD-NA)	Excluded for dementia, moderate/severe depression and anxiety	Medication-OFF; No DBS	Voxel *P* < 0.001, cluster P < 0.05, corrected	3 T MRI	MNI	VMHC	2
Gilmour et al. (2024)	25/28	61.6 (11.3)	LARS	Comparison (PD-A-PD-NA)	Excluded for dementia and major depressive disorder	Medication-ON; No DBS	*P* < 0.05, FWE corrected	3 T MRI	MNI	Task-fMRI	16
Theis et al. (2023)	21/33	70.0 (10)	AES	Correlation	Excluded for dementia; Depression measured (higher in Apathy+)	Medication-OFF; No DBS	*P* < 0.05, FWE corrected	3 T MRI	MNI	GMV	2
Baggio et al. (2015)	25/37	65.60 (12.89)	AS	Comparison (PD-A-D-NA) correlation	Excluded for dementia; Depression and cognition as covariates	Medication-ON; No DBS	*P* < 0.05, FDR corrected	3 T MRI	MNI	FC	11
Zhang et al. (2024)	27/37	61.63 (10.58)	AS	Comparison (PD-A-PD-NA)	Excluded for dementia, depression and anxiety	Medication-OFF; No DBS	*P* < 0.05, FWE corrected	3 T MRI	MNI	FC	1

AS, Apathy Scale; AES, apathy evaluation scale; LARS, Lille apathy rating scale; PD-A, Parkinson Disease with apathy; FrSBE, frontal systems behavior scale; NPI-A, neuropsychiatric inventory-apathy subscale; SAS, Starkstein apathy scale; DBS, Deep brain stimulation; UPDRS-I: Unified Parkinson’s Disease Rating Scale-I; PD-NA, Parkinson Disease without apathy; ALFF, amplitude of low frequency fluctuations; FC, functional connectivity; GMV, gray matter volume; GMD, gray matter density; VMHC, voxel-mirrored homotopic connectivity; ReHo, regional homogeneity; PET, positron emission tomography; MNI, Montreal Neurological Institute; MRI, Magnetic Resonance Imaging; fMRI, functional magnetic resonance; FDR, False discovery rate; FWE, Family wise error.

### Convergent FC correlates of PD-A

**Figure 1:**
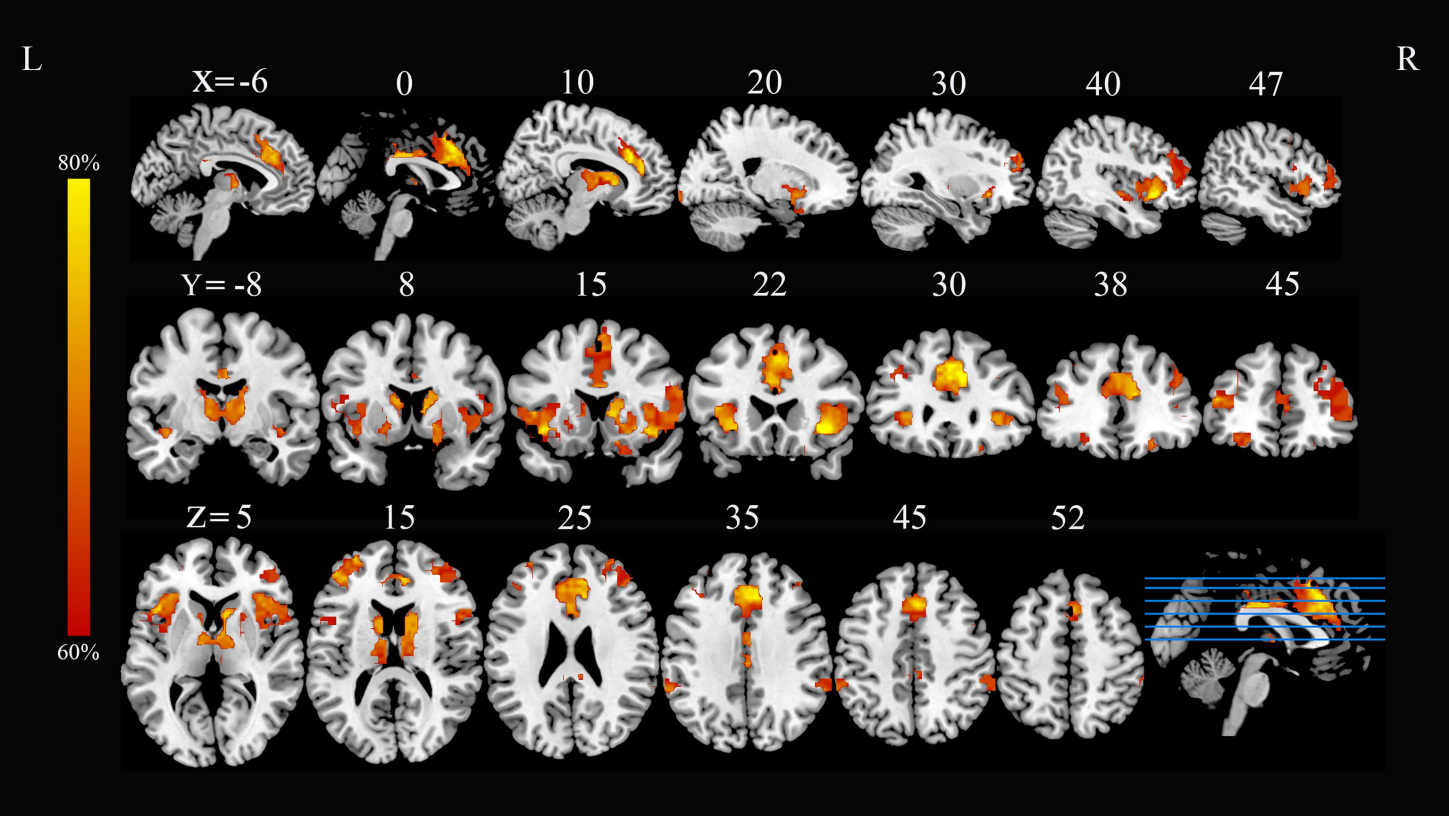
PD-A-associated FC overlap maps based on 4 mm radius sphere. Dysfunctional brain networks are shown as FC probability maps thresholded at 60%, showing brain regions functionally connected to more than 60% of the contrast seeds. PD-A, Parkinson’s disease with apathy; FC, functional connectivity; L, left; R, right.

Application of the FCNM approach, integrating coordinates of reported functional and structural correlates of PD-A from the 24 studies with the normative HCP connectome, revealed convergent FC associated with PD-A. These FC patterns involved widely distributed brain regions, primarily including the bilateral inferior frontal gyrus, bilateral anterior insula, bilateral dorsolateral prefrontal cortex (DLPFC), bilateral caudate nucleus, and bilateral thalamus (*p* < 0.05, FDR corrected) (Figure 1).

### Association with canonical brain networks

**Figure 2:**
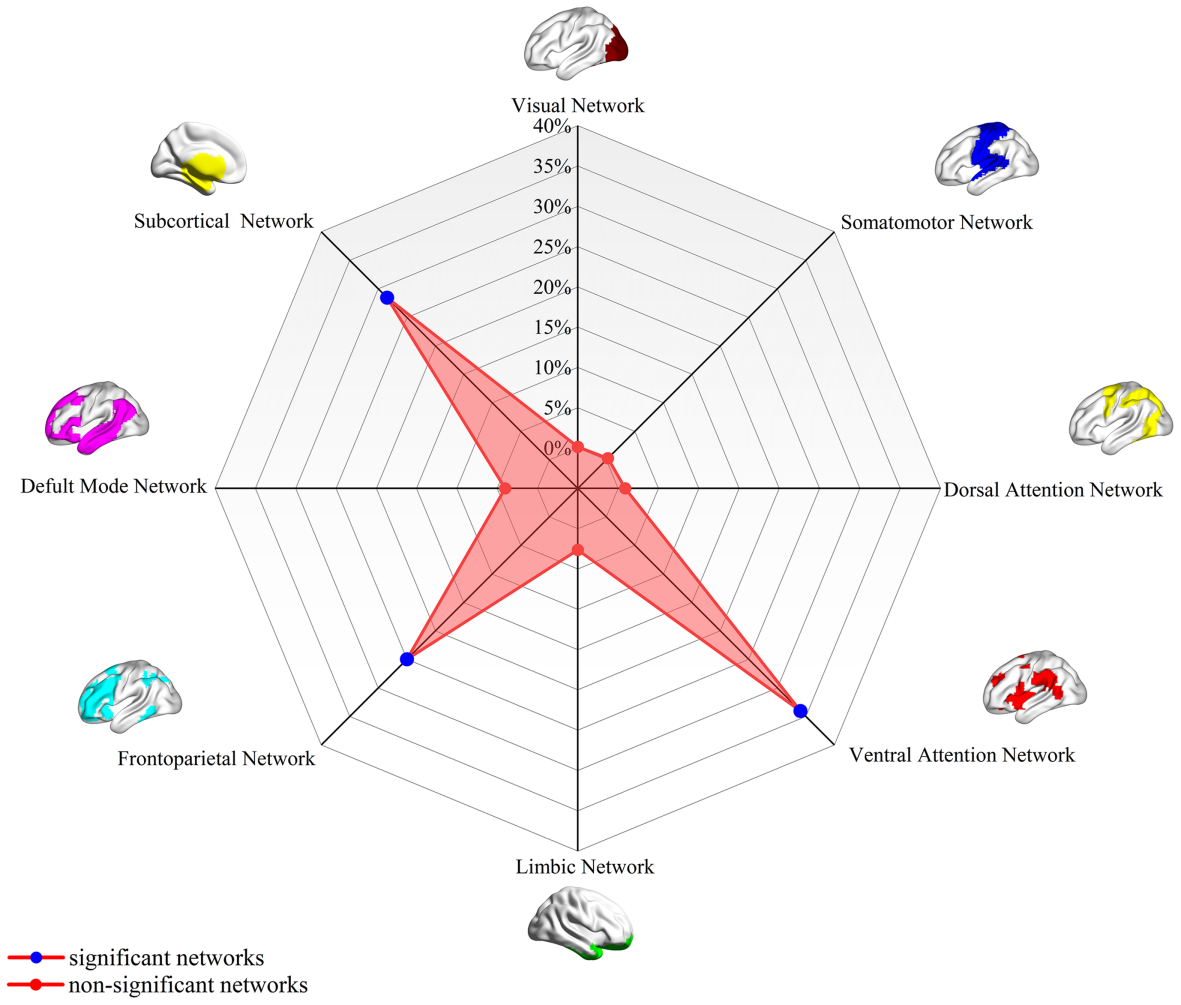
PD-A-associated FC overlap maps based on 4-mm radius sphere in association with canonical brain networks. Polar plots illustrate the proportion of overlapping voxels between each PD-A FC map and a canonical network to all voxels within the corresponding canonical network. Note: The blue dot represents brain dysfunction networks, defined as significant networks, exhibiting ≥10% overlap with canonical networks, whereas the red dot represents non-significant networks with <10% overlap. PD-A, Parkinson’s disease with apathy; FC, functional connectivity.

Analysis of the spatial overlap between the PD-A-related aberrant FC and established canonical brain networks indicated preferential involvement of specific systems. The network showed the highest overlap with the VAN, largely corresponding to the b bilateral inferior frontal gyrus and bilateral anterior insula (overlap proportion: 34.05%). Significant overlap was also observed with the FPN, primarily involving bilateral DLPFC (overlap proportion: 24.89%). Additionally, significant overlap was also observed with the subcortical network, primarily involving bilateral caudate nucleus and bilateral thalamus (overlap proportion: 28.47%) (Figure 2). Overlap proportions with the remaining canonical networks (visual, SMN, DAN, limbic network, and DMN) were all below 10%.

### Robustness analyses

FCNM analyses repeated using seed spheres with 1-mm and 7-mm radii yielded topographically similar network patterns to those obtained using the standard 4-mm radius sphere (Supplementary Figures S2, S3, respectively). Furthermore, the pattern of canonical network involvement remained consistent when replicating the FCNM procedure with spheres of 1-mm and 7-mm radii (Supplementary Figures S4, S5, respectively).

## Discussion

To our knowledge, this study is the first to integrate FCNM with large-scale human brain connectome data from the HCP to demonstrate that heterogeneous functional and structural correlates of apathy in PD map onto common brain networks. Our analysis revealed that aberrant brain regions previously identified in the literature indeed map onto a specific network encompassing key nodes within the VAN (notably the bilateral inferior frontal gyrus and bilateral anterior insula), FPN (bilateral DLPFC), and subcortical network (bilateral caudate nucleus and bilateral thalamus). Importantly, these findings demonstrated high robustness, with the primary network localization (derived from 4-mm radius seed spheres) being consistently replicated in validation analyses using both 1-mm and 7-mm radii.

A major finding of our study was the significant mapping of PD-A associated functional and structural alterations onto the subcortical network. The subcortical network, primarily comprising the basal ganglia, thalamus, amygdala, and select cerebellar nuclei (Fan et al., 2016), is essential for the regulation of motor control, emotion, and cognition (Zhou et al., 2024). Converging evidence suggests that the neural underpinnings of PD-A can be conceptualized as a multi-tiered mechanism of functional deterioration that initiates in subcortical areas and leads to widespread network disintegration (Hanganu et al., 2014; Gorges et al., 2015). The emergence of PD-A is increasingly attributed to the disruption of fronto-striatal-limbic circuitry, which critically undermines the neural computations for reward valuation, emotional processing, and goal-directed behavior (Moretti and Signori, 2016; Wen et al., 2016). Driven by dopaminergic degeneration, this circuitry undergoes a progression of functional inactivation that precedes structural atrophy (Erro et al., 2020). Early evidence indicates that key nodes within this circuit—such as the caudate nucleus—exhibit reduced FC (Baggio et al., 2015) and hypoperfusion (Erro et al., 2020). As the disease progresses, this is compounded by the microstructural disruption of connecting fiber tracts (Prange et al., 2019) and GM alterations (Maggi et al., 2024). Crucially, this internal disintegration leads to the functional failure of the motivation network as a “network hub,” disrupting effective information exchange with the cortical executive control network (Costa et al., 2018). Specifically, the “bottom-up” transmission of motivational and emotional signals is impeded (Zeng et al., 2023), leaving the brain’s decision-making systems without effective drive. Thus, apathy in PD should not be viewed as an isolated symptom, but as the end point of a pathological process in which the subcortical network collapses and becomes isolated from external networks, leading to profound deficits in goal-directed behavior (Alzahrani et al., 2016; Dujardin et al., 2007).

In our study, the caudate nucleus and thalamus emerged as critical dysfunctional hubs within the subcortical network linked to PD-A. The caudate nucleus, a crucial element of the basal ganglia, governs motor control, learning and memory, reward and motivation, and executive functions (Barrett et al., 2024). Numerous neuroimaging and neurotransmitter studies have shown that individuals with PD-A frequently display diminished GM volume, lower FC, and compromised dopaminergic transmission within the caudate nucleus (Prange et al., 2019; Wen et al., 2016; Carriere et al., 2014; Santangelo et al., 2015; Martinez-Horta et al., 2017). These structural and functional abnormalities lead to the disruption of reward-motivation circuits, which in turn contributes to symptoms of apathy, such as loss of interest and lack of initiative (Béreau et al., 2023; Thobois et al., 2017). Consequently, pathology of the caudate nucleus represents one of the core neural underpinnings for the development of PD-A, highlighting its significance for early identification and intervention (Martinez-Horta et al., 2017; Lucas-Jiménez et al., 2018; Camargo et al., 2018; Sun et al., 2020). The thalamus plays a crucial role as a central hub and gateway in the cortico-striato-thalamo-cortical (CSTC) circuit, overseeing the regulation of motivation, planning, and goal-directed behavior by transmitting signals from the basal ganglia to the prefrontal cortex (Béreau et al., 2023; Haber and Calzavara, 2009; Sherman, 2016). Although one study found an association between apathy and atrophy of the mediodorsal thalamus (Moretti et al., 2012), other studies have reported that such atrophy is associated with the co-occurrence of apathy and impulse control disorders rather than with pure apathy (Maggi et al., 2024), or have found no significant atrophy in apathetic groups (Lucas-Jiménez et al., 2018). Furthermore, the severity of apathy is closely associated with significantly reduced metabolic activity in the thalamus (particularly its medial and ventral portions) (Drapier et al., 2006; Del-Monte et al., 2017), and the root of this functional decline lies in the disruption of structural and FC between the thalamus and its upstream and downstream partners in the motivation circuit, such as the prefrontal cortex (Lucas-Jiménez et al., 2018). The underlying cause of this network disruption is dopaminergic dysfunction in the mesolimbic system, which directly impairs the thalamus’s ability to process and transmit signals, making it a key anatomical target of the “hypodopaminergic syndrome” and ultimately blocking the effective upstream transmission of motivational signals, leading to the clinical manifestation of apathy (Skorvanek et al., 2015).

Our study also found a significant convergence of PD-A-related network alterations on the FPN. The FPN, as a core hub of higher-order cognitive functions in the human brain, is primarily responsible for the generation and maintenance of motivated behaviors, including motivational regulation, goal setting, attention control, behavioral planning, and execution (Ducharme et al., 2018; Gao and Wu, 2016; Yau et al., 2018). Accumulating evidence shows that both functional and structural disruption of the FPN constitutes a principal neurobiological substrate of apathy in PD (Ducharme et al., 2018; Prodoehl et al., 2014; Peralta et al., 2019; Devignes et al., 2022). On the functional level, PET studies have demonstrated reduced glucose metabolism in frontoparietal regions (Prodoehl et al., 2014; Peralta et al., 2019), while fMRI research has revealed weakened intra-network connectivity and impaired information integration between networks within the FPN—findings closely related to the occurrence of apathy (Ducharme et al., 2018; Gao and Wu, 2016). Structurally, the severity of apathy correlates significantly with GM atrophy in the frontoparietal cortex and with decreased integrity of the white matter tracts connecting key FPN nodes (Yau et al., 2018; Devignes et al., 2022). Disruption of this network may impair behavioral initiative and motivation, thus providing a mechanistic explanation for apathy in PD and a foundation for improved diagnosis and intervention.

The DLPFC, a key FPN node, is involved in higher-order cognitive functions such as working memory, attentional control, goal-directed planning, decision-making, and self-monitoring (Tan et al., 2023; Godet et al., 2024). In the context of apathy associated with PD, abnormalities in the function and structure of the DLPFC are widely considered a critical neural basis for this symptom (Wen et al., 2016; Kostić and Filippi, 2011). Numerous structural and functional neuroimaging studies have found that PD patients with apathy commonly exhibit reduced GM volume in the DLPFC (Martinez-Horta et al., 2017), and decreased metabolism (Raimo et al., 2019). These impairments directly affect the generation and maintenance of goal-directed behavior, leading to clinical manifestations of apathy, such as a lack of motivation and reduced self-initiation (Sinha et al., 2013). This functional insufficiency arises from disrupted signaling in fronto-subcortical pathways that support DLPFC function, driven by degeneration of critical neurotransmitter systems including dopamine and acetylcholine (Santangelo et al., 2015; Sperling et al., 2023).

Our findings further indicate significant mapping of PD-A-associated abnormalities onto the VAN. The VAN comprises the temporoparietal junction and ventral frontal cortex, and is typically implicated in stimulus-driven attentional control (Vossel et al., 2014). Numerous neuroimaging and clinical studies have demonstrated that structural and functional alternations of the VAN are closely associated with the development of apathy (Wu et al., 2024; Dolphin et al., 2023). In various neurological disorders such as Alzheimer’s disease and frontotemporal dementia, patients with apathy often exhibit GM atrophy, reduced metabolism, and weakened network connectivity in VAN-related regions (Jenkins et al., 2022; Tumati et al., 2020). These deficits lead to a diminished capacity for motivational processing and a reduced sensitivity to environmental and emotional stimuli, manifesting as the classic symptoms of apathy, such as diminished initiative, loss of interest, and emotional blunting (Dolphin et al., 2023). Our analyses further identified the anterior insula and inferior frontal gyrus as pivotal nodes of dysfunction within the VAN implicated in PD-A. The anterior insula serves as a crucial integrative hub in the human brain, merging interoceptive signals with emotional and cognitive information to generate subjective emotional awareness (Christopher et al., 2014; Starkstein and Brockman, 2018). In the context of PD, Substantial evidence shows that PD patients with apathy exhibit definite pathological changes in the anterior insula, including structural GM atrophy, microstructural white matter tract disruption, and abnormal metabolic activity (Robert et al., 2012; Raimo et al., 2019; Jonkman et al., 2021).

Within the VAN, inferior frontal gyrus is a primary component of the prefrontal cortex and is involved in emotional regulation and processing (Li et al., 2020). Together with the basal ganglia, thalamus, and limbic system, it forms an integral part of the fronto-striato-limbic motivational circuits, which are essential for maintaining individual initiative and internal drive (Kostić and Filippi, 2011). In patients with PD-A, a substantial body of neuroimaging and neuropsychological evidence has revealed that the inferior frontal gyrus undergoes GM volume reduction and structural atrophy (Alzahrani et al., 2016), decreased glucose metabolism (Robert et al., 2014), and weakened FC with other nodes within these circuits (Baggio et al., 2015; Lucas-Jiménez et al., 2018). Crucially, these abnormalities are all significantly correlated with the severity of apathy symptoms. Together, dysfunction of the inferior frontal gyrus and anterior insula represents a fundamental neurobiological basis for apathy in PD, highlighting their potential as key targets for early diagnosis and intervention.

Taken together, our findings pinpoint the DLPFC and IFG as readily targetable nodes for neuromodulatory interventions like transcranial magnetic stimulation. Indeed, the clinical relevance of these targets is underscored by their well-established efficacy in ameliorating symptoms of PD (Pal et al., 2010; Bhat et al., 2023; Srovnalova et al., 2011). This provides a compelling rationale for a network-based therapeutic strategy: stimulating these sites may not only alter local neuronal activity but also globally rebalance the apathy network, potentially unlocking greater clinical gains than conventional single-target approaches. Prospectively, the connectivity profile between a patient’s deep brain stimulation electrode placement and this network could offer significant clinical utility, serving to predict treatment efficacy or stratify the risk of apathy as an adverse outcome.

### Limitations

Several limitations should be considered when interpreting the findings of this study. First, the analysis relies on coordinate-based data extracted from published studies, which represents a summary statistic (peak location) and inherently loses spatial information compared to analyses using full statistical maps or individual participant data (Darby et al., 2019; Wang et al., 2024; Cheng et al., 2025; Stubbs et al., 2023). This coordinate-based approach is correlational and cannot establish causality between the identified network and PD-A related functional and structural alterations. Second, we utilized a normative connectome derived from healthy adults (HCP dataset) to map the networks associated with PD-A functional and structural alterations. While this is standard practice in network mapping (Darby et al., 2019; Xu et al., 2024; Cheng et al., 2025; Fox et al., 2014) and evidence suggests sample selection often has minimal impact on the topography of network localization (Boes et al., 2015; Fox et al., 2014; Horn et al., 2017), using a connectome derived from a sample more closely matched to the PD-A populations might potentially yield slightly different results, though previous work suggests topographic differences are often minor (Stubbs et al., 2023; Horn et al., 2017; Joutsa et al., 2022). Future research using large-scale, age-matched, or disease-specific connectomes will be instrumental in further validating the present findings Third, the input coordinates were derived from studies employing diverse imaging modalities (PET, structural MRI, resting-sate fMRI, task-fMRI) and analytical techniques (e.g., GMV, task activation, various resting-sate fMRI metrics). While FCNM is designed to identify convergence despite such heterogeneity, these variations inevitably introduce noise and could potentially obscure more subtle or specific network effects. Specifically, the number of included studies was insufficient to permit robust subgroup analyses based on imaging modality, which prevented us from exploring potentially distinct network patterns associated with functional versus structural alterations. Furthermore, the original studies varied in their diagnostic criteria for apathy and PD, sample sizes, and control for potential confounds (e.g., cognitive impairment, depression, medication status), which could also contribute to the heterogeneity of input data. Fourth, methodological choices such as the radius of the coordinate spheres (though sensitivity analyses showed robustness) and the probability threshold (60%) for defining the final network represent specific parameters (Darby et al., 2019; Burke et al., 2020). Exploring alternative parameters could provide further insights but was beyond the scope of this initial network localization. Fifth, the current analysis did not incorporate negative findings from the original studies, nor was it feasible to weight studies based on quality metrics or sample size beyond the initial selection criteria. The potential influence of publication bias, which tends to favor positive findings, cannot be entirely discounted. Sixth, a significant limitation of this study was the small number of included studies that employed a strict definition of pure apathy, which precluded a robust subgroup analysis of the influence of comorbidities. Seventh, A core limitation of this study is the significant heterogeneity of the primary literature, which in turn constrained our analytical approach. The variability in diagnostic criteria for apathy across studies forced our reliance on a normative young-adult connectome-a necessary simplification that does not model age- or disease-specific network changes. As a result, while the identified network provides a promising basis for clinical translation, its proposed applications in guiding TMS/DBS targeting should be considered a preliminary hypothesis that requires rigorous prospective validation. Finally, while our network localization approach provides a unifying framework, the identified network represents regions functionally connected to the sites of reported alterations in PD-A. The precise pathophysiological mechanisms linking these network components to the multifaceted symptoms of apathy in PD warrant further investigation, ideally through prospective studies combining multimodal neuroimaging, detailed clinical phenotyping, and longitudinal assessments.

## Conclusion

In summary, our findings indicate that brain functional and structural correlates of apathy in PD converge on distributed networks involving the VAN, FPN, and subcortical circuits. The identification of network involvement underscores the interplay between motivational, executive, and attentional processes in the manifestation of apathy, emphasizing the multisystemic nature of this syndrome. This network localization approach offers a comprehensive and unified framework that may help reconcile inconsistencies in previous studies investigating the neurobiological underpinnings of PD-A and may improve the reproducibility and translational potential of research in this area. Future research should now focus on clinically validating this network as a predictive biomarker and therapeutic target through large-scale, standardized studies employing disease-specific connectomes.

## Data Availability

The original contributions presented in the study are included in the article/Supplementary material, further inquiries can be directed to the corresponding author.

## Footnotes

1
https://www.sdmproject.com/utilities/?show=Coordinates

2
https://www.fil.ion.ucl.ac.uk/spm/

3
https://rfmri.org/DPABI

## Author’s note

All authors approved the final version of the manuscript being submitted. This manuscript is the authors’ original work, had not been under review elsewhere, and its findings have neither been published previously nor accepted for publication.
