# Supplementary material for: Network localization of functional and structural correlates of apathy in Parkinson’s disease
Source: Front Syst Neurosci. 2026 Mar 3;20:1724421. doi: 10.3389/fnsys.2026.1724421 (PMC12992048; doi:10.3389/fnsys.2026.1724421)
Supplement: Supplementary file 1 [file Data_Sheet_1.docx]

**Supplementary materials**

**Network localization of** **functional and structural correlates of apathy in Parkinson’s disease**

**Keywords for literature search**

((Parkinson’s disease) OR parkinson*)) AND ((apath*)) AND ((magnetic resonance imaging) OR (neuroimaging) OR (MRI) OR (resting state functional MRI) OR (rs-fMRI) OR (brain connectivity) OR (FC) OR (functional connectivity) OR (ReHo) OR (regional homogeneity) OR (ALFF) OR (amplitude of low frequency fluctuations) OR (fALFF) OR (fractional amplitude of low-frequency fluctuations) OR (low frequency fluctuation) OR (LFF) OR (cerebral blood flow) OR (CBF) OR (arterial spin labeling) OR (ASL) OR (independent component analysis) OR (ICA) OR (degree centrality) OR (DC) OR (VMHC) OR (voxel-mirrored homotopic connectivity) OR (PET) OR (positron emission tomography) OR (SPECT) OR (single photon emission computed tomography) OR (structural magnetic resonance imaging) OR (morphometry) OR (voxel-based) OR (voxel-wise) OR (voxel-based morphometry) OR (VBM) OR (structural neuroimaging) OR (GMV) OR (grey matter) OR (gray matter))

**Table S1. Demographic information of the HCP**

| **Dataset sample size** | **Age (years)** | **Gender (F/M)** |
| --- | --- | --- |
| HCP 1093 | 28.78±3.69 | 594/499 |

Age is expressed as mean ± standard deviation. Note: HCP, Human Connectome Project; F, female; M, male.

**Table S2. Resting-state fMRI parameters of the HCP**

| **Parameter** | **HCP** |
| --- | --- |
| Scanner | 3.0T Siemens Trio |
| Sequence | GRE-EPI |
| TR (ms) | 720 |
| TE (ms) | 33.1 |
| FA (°) | 52 |
| FOV (mm²) | 208×180 |
| Matrix size | 104×90 |
| Slice thickness (mm) | 2 |
| Slice gap (mm) | 0 |
| Slices | 72 |
| Time points | 1210 |

HCP, Human Connectome Project; GRE-EPI, gradient-recalled echo-Planar Imaging; FA, flip angle; fMRI, functional magnetic resonance imaging; FOV, field of view; TE, echo time; TR, repetition time.


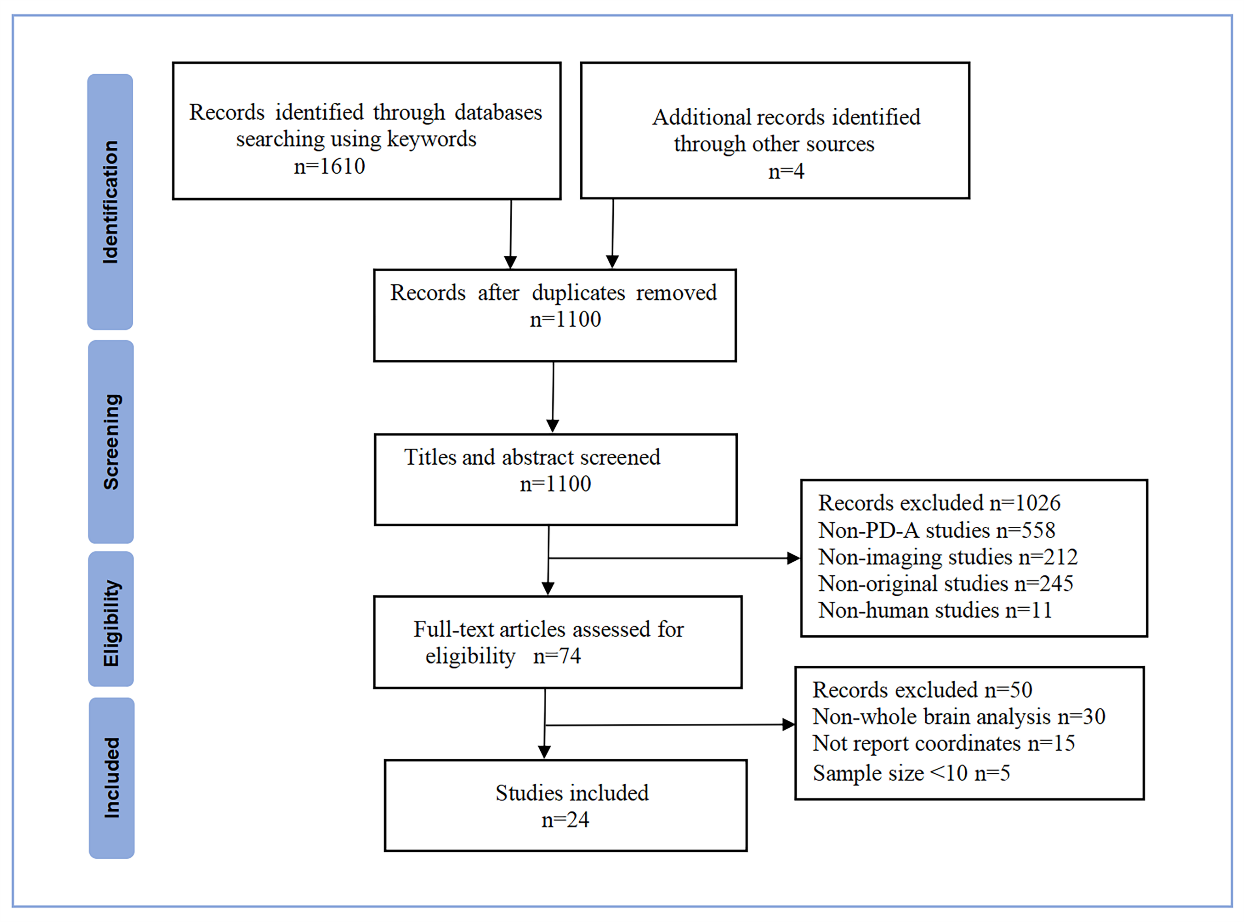


**Figure S1. Flow chart showing the process of study identification and exclusion**

PD-A, Parkinson’s disease with apathy


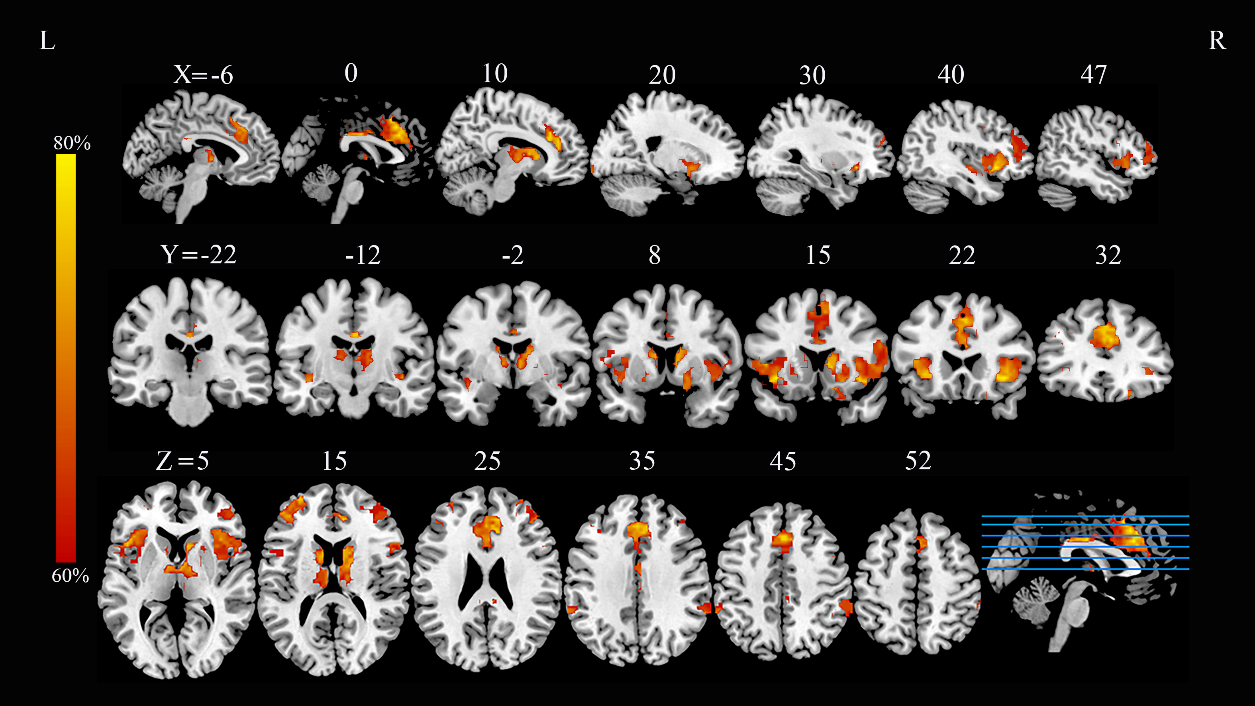


**Figure S2. PD-A-associated FC overlap maps based on 1-mm radius sphere**

Dysfunctional brain networks are shown as FC probability maps thresholded at 60%, showing brain regions functionally connected to more than 60% of the contrast seeds. PD-A, Parkinson’s disease with apathy; FC, functional connectivity; L, left; R, right


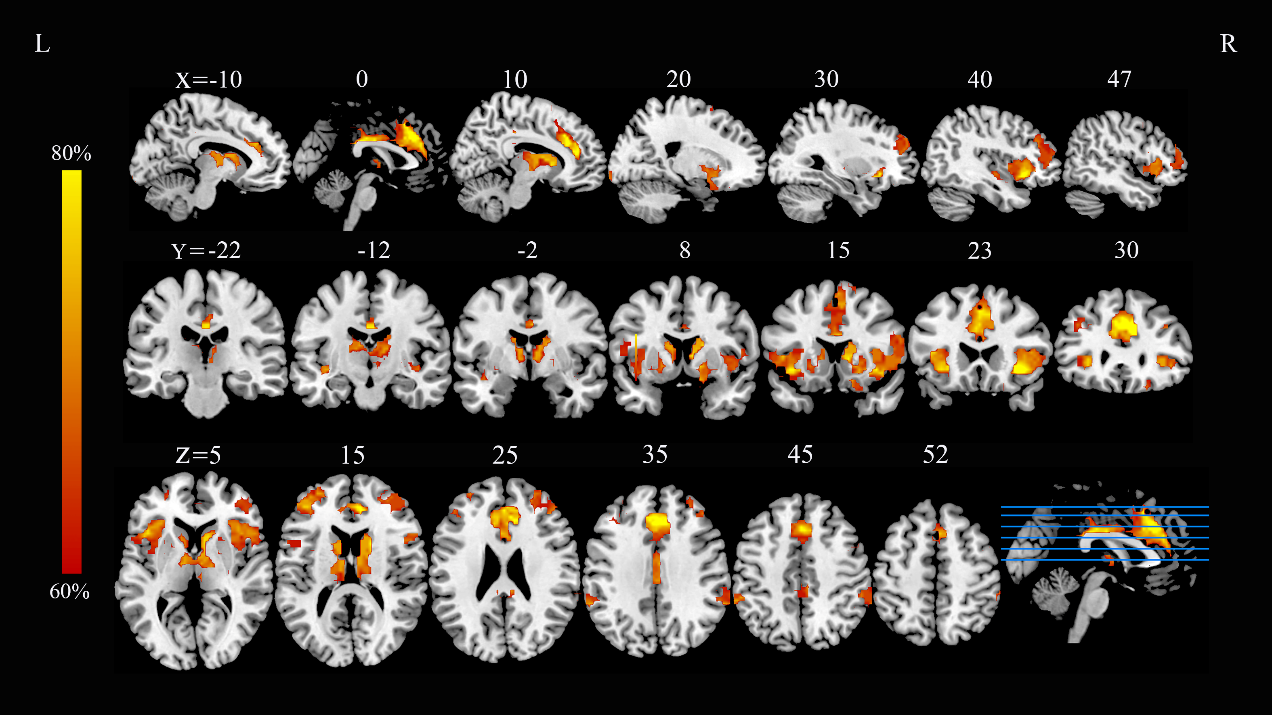


**Figure S3. PD-A-associated FC overlap maps based on 7-mm radius sphere**

Dysfunctional brain networks are shown as FC probability maps thresholded at 60%, showing brain regions functionally connected to more than 60% of the contrast seeds. PD-A, Parkinson’s disease with apathy; FC, functional connectivity; L, left; R, right


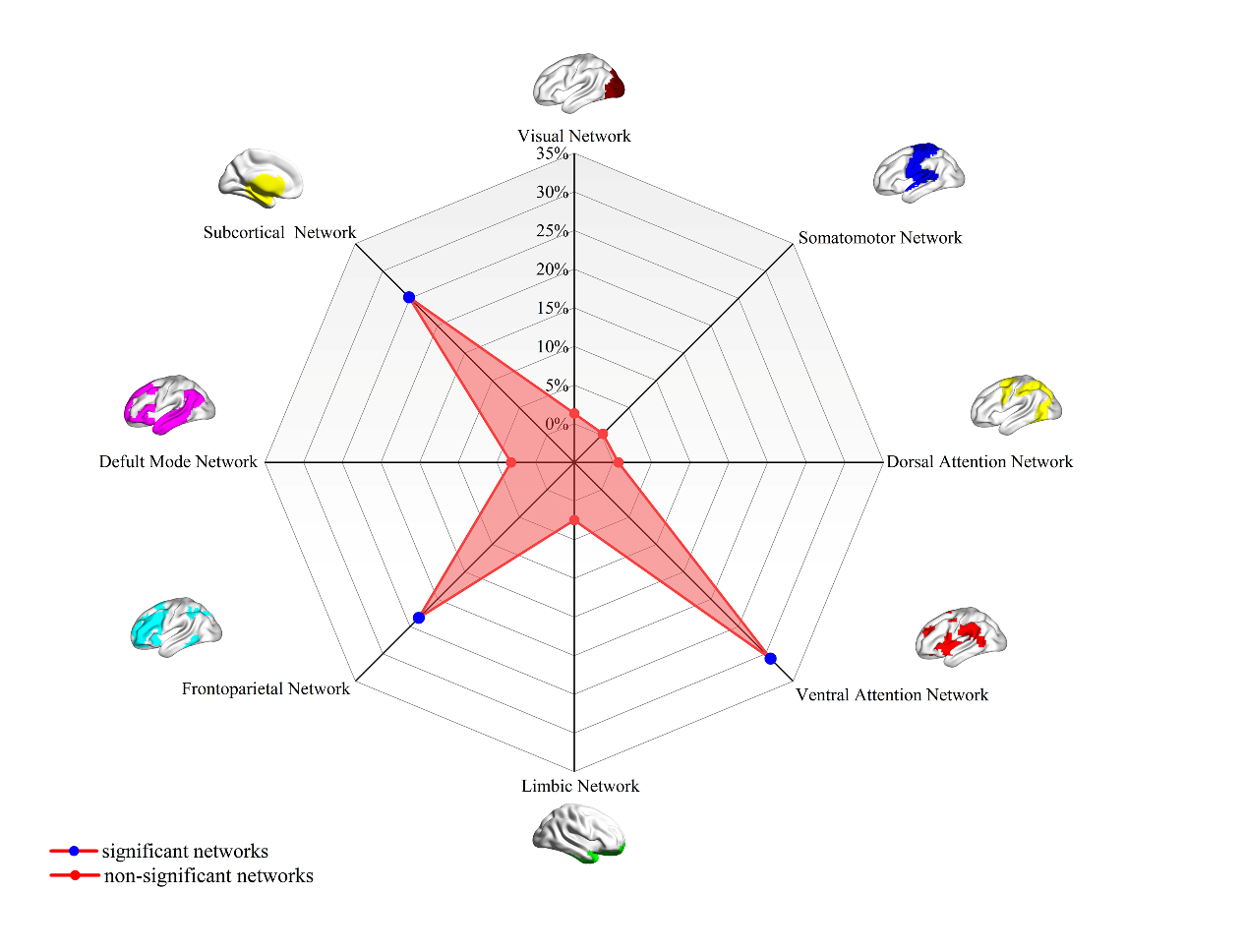


**Figure S4. PD-A-associated** **FC overlap maps based on 1-mm radius sphere in association with canonical brain networks**

Polar plots illustrate the proportion of overlapping voxels between each PD-A FC map and a canonical network to all voxels within the corresponding canonical network. Note: The blue dot represents brain dysfunction networks, defined as significant networks, exhibiting ≥10% overlap with canonical networks, whereas the red dot represents non-significant networks with <10% overlap.

PD-A, Parkinson’s disease with apathy; FC, functional connectivity


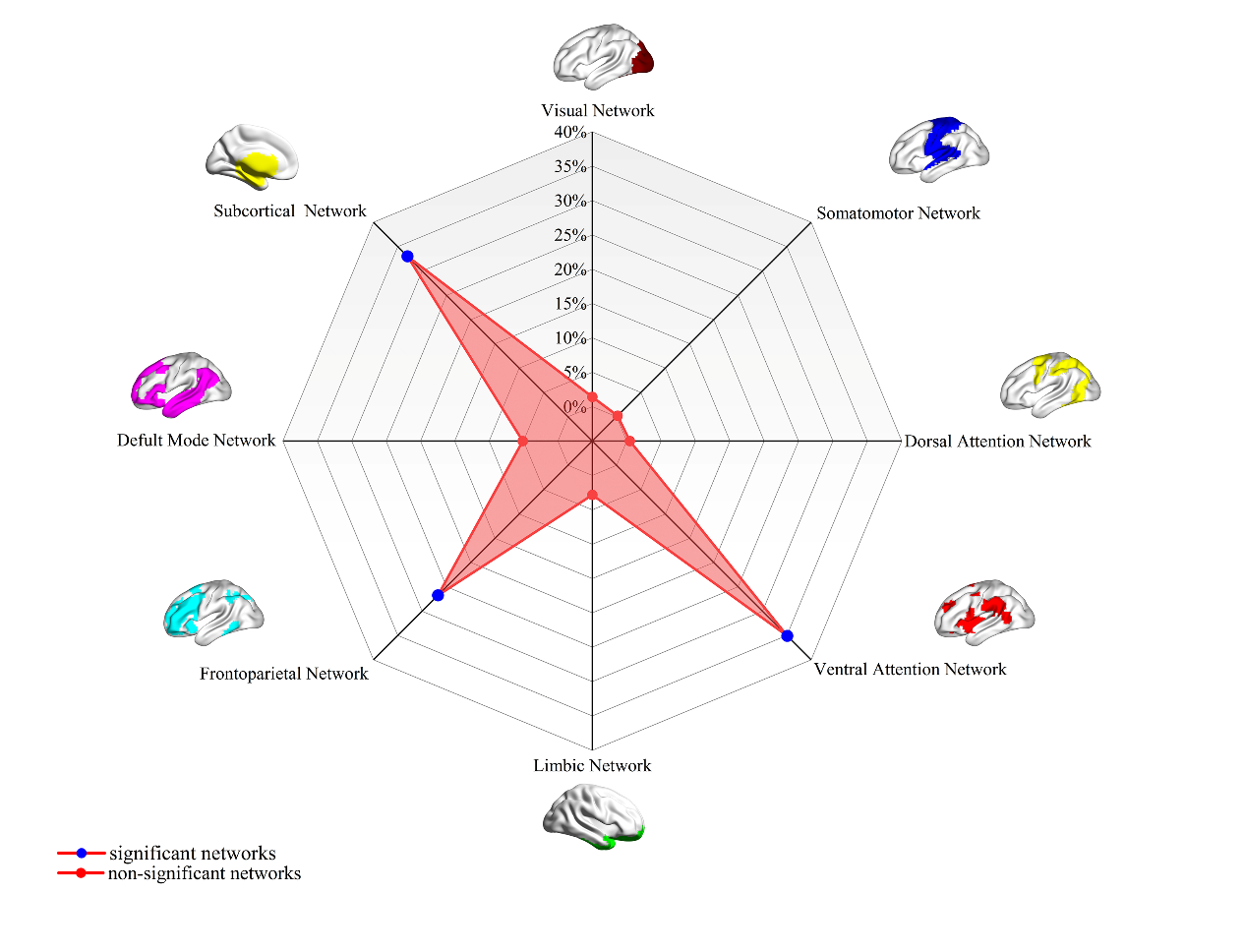
 **Figure S5. PD-A-associated FC overlap maps based on 7-mm radius sphere in association with canonical brain networks**

Polar plots illustrate the proportion of overlapping voxels between each PD-A FC map and a canonical network to all voxels within the corresponding canonical network. Note: The blue dot represents brain dysfunction networks, defined as significant networks, exhibiting ≥ 10% overlap with canonical networks, whereas the red dot represents non-significant networks with <10% overlap.

PD-A, Parkinson’s disease with apathy; FC, functional connectivity
